# Pathogenicity and virulence of *Streptococcus pneumoniae*: Cutting to the chase on proteases

**DOI:** 10.1080/21505594.2021.1889812

**Published:** 2021-03-04

**Authors:** Mary E. Marquart

**Affiliations:** Department of Microbiology and Immunology, University of Mississippi Medical Center, Jackson, Mississippi USA

**Keywords:** Peptidase, protease, proteinase, *Streptococcus pneumoniae*, virulence

## Abstract

Bacterial proteases and peptidases are integral to cell physiology and stability, and their necessity in *Streptococcus pneumoniae* is no exception. Protein cleavage and processing mechanisms within the bacterial cell serve to ensure that the cell lives and functions in its commensal habitat and can respond to new environments presenting stressful conditions. For *S. pneumoniae*, the human nasopharynx is its natural habitat. In the context of virulence, movement of *S. pneumoniae* to the lungs, blood, or other sites can instigate responses by the bacteria that result in their proteases serving dual roles of self-protein processors and virulence factors of host protein targets.

## Pathogenesis: Colonization, establishment of disease, and immune subversion

*Streptococcus pneumoniae* (pneumococcus) is carried in the human nasopharynx of a varied percentage of individuals (moreso in children) and can maintain residence there, or spread to other body sites and cause opportunistic infections. The commonly known diseases caused by *S. pneumoniae* are pneumonia, otitis media, meningitis, and septicemia, although these diseases are not a comprehensive list. Transmission of pneumococci between individuals occurs through close contact and aerosols, and colonization is considered a prerequisite for disease although many colonized individuals do not experience symptoms. Therefore, the ability of *S. pneumoniae* to adhere to nasopharyngeal mucosal epithelial cells is an important step in the process leading to pathogenesis. Pneumococcal factors shown to play roles in host cell attachment are surface proteins such as pneumococcal surface adhesin A (PsaA) and choline-binding protein A/pneumococcal surface protein C/*S. pneumoniae* secretory IgA binding protein (CbpA/PspC/SpsA). PsaA binds to host E-cadherin [1] and CbpA/PspC/SpsA binds to sialic acid, lacto-N-neotetraose, the polymeric immunoglobulin (Ig) receptor, and vitronectin [2–4].

The next general step in pneumococcal pathogenesis is establishment of the bacteria in the lungs, blood, middle ear, central nervous system, or other site. The mode of this establishment depends on host susceptibility, regulation of bacterial gene expression, and the opportunity for interactions between *S. pneumoniae* and host components. In the case of pneumococcal pneumonia, pneumococcal neuraminidase (NanA) is essential for cleavage of sialic acid from host cell glycoprotein receptors, thus promoting attachment of *S. pneumoniae* to airway epithelial cells [5,6]. Pneumococcal pneumonia is characterized by lung inflammation as a result of bacterial factors eliciting pro-inflammatory cytokine responses and immune cell recruitment. The cholesterol-dependent cytolysin, pneumolysin, contributes to these inflammatory effects in addition to its role of forming pores in eukaryotic cell membranes [7,8].

Regardless of whether *S. pneumoniae* remains confined to the lung or spreads to the blood to cause septicemia, the bacteria are confronted with various host defenses. Pneumococci utilize strategies to escape or modify the immune response to survive. For instance, when bound to the mannose-receptor C type 1 on dendritic cells or alveolar macrophages, pneumolysin promotes uptake of *S. pneumoniae* and subsequent escape from lysosomes [9]. In the blood, pneumococcal surface protein A (PspA) and the outer polysaccharide capsule contribute to bacterial survival. One of the contributions of PspA is to shortcircuit opsonization of pneumococci by inhibiting complement C3 deposition [10]. The capsule aids in protection from phagocytosis by masking pneumococcal surface antigens from C3 [11,12], and reduces pneumococcal enmeshment in neutrophil extracellular traps [13].

Discovery of functions not previously ascribed to well-known pneumococcal virulence factors underscores the fact that, despite pneumococcal pathogenesis having been studied for over 100 years, new information regarding *S. pneumoniae* virulence factors and mechanisms as well as host responses continues to be uncovered. Shifting trends in epidemiology, longer life expectancies, human crowding, and strain selection caused by antibiotic use and pneumococcal vaccination contribute to new information. Additionally, advancements in molecular, biochemical, and bioinformatic methods have revolutionized the understanding of pneumococcal pathogenesis. One of the topical gaps in our knowledge that has the potential for providing key information regarding the virulence of *S. pneumoniae* is the protease.

## Proteases

Proteases are enzymes that cleave proteins and peptides, and are ubiquitous in the different kingdoms of life. Generally, these enzymes either hydrolyze peptide bonds within, or cut the bonds near the amino- or carboxyl-terminal ends, of proteins. Their functions are wide-ranging and include processing or degradation of improperly folded proteins and preparation of pre-proteins for secretion by signal cleavage. Bacterial proteases play key roles in cell homeostasis, protein transport, and cell wall structural integrity. In the context of pathogenesis, bacterial proteases have been documented to possess key functions such as severing mammalian host immune components, thus rendering them inactive, or degrading host cell membrane proteins and damaging tissues. As increasing numbers of bacterial genomes are being annotated and deposited, mining for under- or unstudied putative proteases can complement the established literature on what is already known and prompt novel investigations into their functions.

Certain bacterial species are well-known to produce proteases that play significant roles in pathogenesis. *Pseudomonas aeruginosa* is a causative agent of burn wound infections, pneumonia, and ocular infections to name a few, and produces several proteases implicated in direct damage to the host. For example, elastase B causes extensive damage to the cornea during ocular infection [14] and lung injury during pneumonia [15]. Virulence factor studies of *Streptococcus pneumoniae*, on the other hand, have historically focused heavily on the polysaccharide capsule as an antiphagocytic factor and pneumolysin as a multifunctional toxin and mediator belonging to the family of cholesterol-dependent cytolysins. Cell wall-associated proteins, 13–16 of which are choline-binding proteins [reviewed in 16], have also been studied in the context of adhesion and antigenicity and have shown promise as potential vaccine candidates. Research into *S. pneumoniae* proteases, however, has arguably been less emphasized than other factors until more recently.

In 1973–1974, Johnson demonstrated that *S. pneumoniae* produced a tripeptidase and a dipeptidase which were intracellular or cell-associated and exhibited preferences for methionine residues [17,18]. Five years later, IgA1 protease was identified in *S. pneumoniae* [19,20]. The discovery of other proteases, however, was not highlighted until 1991 when Courtney, who showed that several potential proteases with susceptibilities to different serine protease inhibitors, could cleave fibronectin, elastin, fibrinogen, and laminin [21]. As molecular tools such as next-generation sequencing of bacterial genomes and bioinformatics have progressed over the years, the identification and study of putative pneumococcal proteases has increased and uncovered additional clues as to how this opportunistic pathogen damages host cells/tissues and subverts the immune response. A product of this recent progress is the identification of 34 proteins predicted to be proteases in *S. pneumoniae* TIGR4 using sequence data and protein analysis tools [22]. Herein we aim to provide a current review of *S. pneumoniae* proteases as confirmed or potential virulence factors, exerting their effects either directly on host cells and factors or indirectly via management of bacterial physiology, competence, protein processing, and competition (summarized in Table 1).

**Table 1.** Proteases of *S. pneumoniae.*

Protease/Peptidase Identification (Other Names)	Genome Locus Tag:* D39 (new) D39 (old) D39V TIGR4 (new) TIGR4 (old)	Function	Comments	References
ATP-dependent Zinc Protease, putative^	SPD_RS01695 SPD_0310 SPV_0310 SP_RS01660 SP_0341	Unknown	Possesses domains of unknown function	
CbpD	SPD_RS10730 SPD_2028 SPV_2028 SP_RS11240 SP_2201	Peptidoglycan hydrolysis	CHAP domain	31, 32
CbpG	SPD_RS01935 SPD_0356 SPV_0356 SP_RS01925 SP_0390	Serine protease; cleaves casein and fibronectin	Frameshifted in D39; intact in TIGR4; S1 family peptidase [His-Asp-Ser catalytic triad]	32, 34
ClpP	SPD_RS03495 SPD_0650 SPV_0650 SP_RS03655 SP_0746	Cleaves proteins tagged for degradation (complex with ClpA or ClpX]	S14 peptidase family [Ser-His-Asp catalytic triad)	36; 37, 38, 41–43, 45, 48–52
ComA	SPD_RS00235 SPD_0049 SPV_0049 SP_RS00255 SP_0042	Cleavage of competence-stimulating peptide at Gly-Gly	Peptidase C39 family, ABC transporter peptidase subunit	47
CppA^	SPD_RS06830 SPD_1278 SPV_1278 SP_RS07120 SP_1449	Cleavage of complement C3	Glyoxalase/Bleomycin Resistance Protein/Dihydroxybiphenyl Dioxygenase homologous superfamily of metalloenzymes	53–57, 98
DacA (D-Alanyl-D-Alanine Carboxypeptidase; Penicillin-binding Protein 3)	SPD_RS04110 SPD_0767 SPV_0767 SP_RS04300 SP_0872	Cleavage of D-alanine residues during peptidoglycan synthesis	Peptidase S11 famly; gene names and synonyms according to D39V annotation: *pbp3, dacA, dacC*	86–88
DacB (LdcB)	SPD_RS02940 SPD_0549 SPV_0549 SP_RS03085 SP_0629	L,D-carboxypeptidase; peptidoglycan hydrolysis	Peptidase M15B family; Hedgehog signaling/DD-peptidase zinc-binding domain superfamily	86, 89–91
FtsH/Yme1/Tma family protein	SPD_RS00060 SPD_0013 SPV_0013 SP_RS00065 SP_0013	ATP-dependent metallopeptidase; Cleaves proteins tagged for degradation	Peptidase M41 carboxyl-terminal domain	
HtpX	SPD_RS06075 SPD_1138 SPV_1138 SP_RS06290 SP_1283	Putative stress response protein; zinc metalloprotease	Peptidase M48 family; contains a conserved zinc-binding HExxH motif found in zinc metalloproteases	
HtrA (DegP)	SPD_RS10945 SPD_2068 SPV_2068 SP_RS11450 SP_2239	Serine protease; chaperone	Peptidase S1C family, protease Do subfamily; PDZ domain [His-Asp-Ser catalytic triad)	59, 60, 62–72
IgA1 Protease (ZmpA)	SPD_RS05470 SPD_1018 SPV_1018 SP_RS05695 SP_1154	Zinc metalloprotease	Contains a conserved zinc-binding HExxH motif found in zinc metalloproteases	19, 20, 73–85
L,D-Carboxypeptidase (Muramoyltetrapeptide Carboxypeptidase)	SPD_RS00960 SPD_0173 SPV_0173 SP_RS00905 SP_0182	Cleavage of the peptidoglycan tetrapeptide	Peptidase family S66	
LepB (Signal peptidase I)	SPD_RS01985 SPD_0367 SPV_0367 SP_RS01995 SP_0402	Serine peptidase; cleaves leader peptide from pro-proteins	Serine-lysine catalytic dyad	107
Lon-like Protease, putative^	SPD_RS09400 SPD_1765 SPV_1765 SP_RS09890 SP_1967	Degradation of misfolded proteins	S16 peptidase family; ATP-dependent serine peptidase	
LspA (Signal peptidase II)	SPD_RS04400 SPD_0819 SPV_0819 SP_RS04590 SP_0928	Cleaves signal sequence from pro-lipoproteins	Peptidase A8, signal peptidase II family; recognizes lipobox sequence	108, 109
Matrixin Family Metalloprotease; Putative Zinc-dependent Protease	SPD_RS02880 SPD_0537 SPV_0537 SP_RS03030 SP_0617	Unknown	Peptidase M10 metallopeptidase family; domain matching the zinc metalloprotease MMP-like subfamily	
M42 Family Metallopeptidase	SPD_RS02930 SPD_0547 SPV_0547 SP_RS03075 SP_0627	Glutamyl aminopeptidase, PepA type, putative	Peptidase M42 family	
M50 Family Metallopeptidase	SPD_RS09330 SPD_1751 SPV_1751 SP_RS09810 SP_1952	Regulated intramembrane proteolysis (RIP] metalloprotease, putative	M50 family of putative membrane-associated zinc metalloproteases; contains a conserved zinc-binding HExxH motif; RIP proteases are involved in a two-step process of cleaving transmembrane proteins; site-2 protease class of zinc metalloproteases	
Methionyl Aminopeptidase (MAP)	SPD_RS05220 SPD_0970 SPV_0970 SP_RS05360 SP_1084	Cleavage of amino-terminal Met residue	Peptidase M24A family of metallopeptidases; cobalt required for activity	
PepA	SPD_RS08780 SPD_1647 SPV_1647 SP_RS09255 SP_1865	Glutamyl aminopeptidase	Peptidase M42 family	58
PepC	SPD_RS01425 SPD_0261 SPV_0261 SP_RS01380 SP_0281	Aminopeptidase	Peptidase C1 family; homologous superfamily of papain-like cysteine peptidases	
PepF (PepB, PepF1)#	SPD_RS04660 SPD_0866 SPV_0866 SP_RS04855 SP_0979	Oligoendopeptidase	Peptidase M3B family	
PepF (PepF2)#	SPD_RS08365 SPD_1571 SPV_1571 SP_RS08835 SP_1780	Oligoendopeptidase	Peptidase M3B family	
PepN	SPD_RS03770 SPD_0700 SPV_0700 SP_RS03900 SP_0797	Lysyl aminopeptidase	M1 family metallopeptidase	23–25
PepO	SPD_RS07765 SPD_1460 SPV_1460 SP_RS08130 SP_1647	Endopeptidase	M13 peptidase family	92, 93, 95–97
PepP (YpdF)	SPD_RS00980 SPD_0177 SPV_0177 SP_RS00925 SP_0187	Aminopeptidase	Described as MP-,MA-,MS-,AP-,NP-specific aminopeptidase YpdF by D39V annotation	
PepQ	SPD_RS07540 SPD_1418 SPV_1418 SP_RS07840 SP_1591	Proline dipeptidase	Peptidase M24 family; creatinase/aminopeptidase P amino-terminal domain	
PepS	SPD_RS01405 SPD_0258 SPV_0258 SP_RS01355 SP_0278	Aminopeptidase	Peptidase M29 family of metallopeptidases	
PepT	SPD_RS04810 SPD_0894 SPV_0894 SP_RS05000 SP_1008	Tripeptide aminopeptidase	Described as a tripeptide aminopeptidase by D39V annotation	
Peptidase C39	SPD_RS10955 – - – - SP_RS02605 – -	Bacteriocin processing, putative	Incomplete in D39	
PepV	SPD_RS02905 SPD_0542 SPV_0542 SP_RS03055 SP_0623	Dipeptidase	Described as most specific for beta-Ala-Xaa by D39V annotation	
PepX (Xaa-Pro Dipeptidyl-Peptidase)	SPD_RS04205 SPD_0787 SPV_0787 SP_RS04410 SP_0894	Cleavage of Xaa-Pro dipeptides (Xaa = a hydrophobic amino acid)	Peptidase S15 family of serine peptidases	
PhtA (PhpA)	SPD_RS05575 SPD_1038 SPV_1038 SP_RS05790 SP_1175	Cleavage of complement C3	Histidine triad HxxHxH motif	53, 98–102
Prp	SPD_RS05320 SPD_0990 SPV_0990 SP_RS05480 SP_1106	Cleavage of bacterial ribosomal protein L27	PrP family of cysteine proteases; putative His-Cys catalytic dyad	
PrsW	SPD_RS00150 SPD_0031 SPV_2079 SP_RS00170 SP_0026	Intramembrane metalloprotease	PrsW family possibly involved in cell membrane stress response; frameshifted in D39	
PrtA	SPD_RS03010 SPD_0558 SPV_0558 SP_RS03145 SP_0641	Serine peptidase; cleavage of leader peptides from lantibiotics	S8 family of lantibiotic leader peptide-processing serine proteases; [Asp-His-Ser catalytic triad sequence]	60, 103–106
PtrB (Protease II)	SPD_RS06300 SPD_1178 SPV_1178 SP_RS06590 SP_1343	Serine peptidase	S9 family peptidase (Ser-Asp-His catalytic triad sequence), usually cleaves oligopeptides	
Pyroglutamyl Peptidase I (Pcp1)	SPD_RS04040 SPD_0753 SPV_0753 SP_RS04215 SP_0860	Cleavage of pyroglutamic acid from peptides or proteins	Peptidase family C15	
Pyroglutamyl Peptidase I (Pcp2)	SPD_RS09930 SPD_1870 SPV_1870 SP_RS10415 SP_2060	Cleavage of pyroglutamic acid from peptides or proteins	Peptidase family C15; frameshifted in D39	
Rhomboid Family Intramembrane Serine Protease	SPD_RS10170 SPD_1920 SPV_1920 SP_RS10665 SP_2094	Intramembrane cleavage of proteins or peptides	Peptidase S54 domain; (Ser-His catalytic dyad)	
RseP (Eep, RasP, RseP, YluC)	SPD_RS01335 SPD_0245 SPV_0245 SP_RS01290 SP_0263	Regulated intramembrane proteolysis [RIP) metalloprotease	M50 family of putative membrane-associated zinc metalloproteases; contains a conserved zinc-binding HExxH motif; PDZ domain; RIP proteases are involved in a two-step process of cleaving transmembrane proteins	33
S8 Family Serine Peptidase	SPD_RS09340 SPD_1753 SPV_1753 SP_RS09820 SP_1954	Serine peptidase; cleavage of leader peptides from lantibiotics	S8 family of lantibiotic leader peptide-processing serine proteases; (Asp-His-Ser catalytic triad sequence]; described as epidermin leader peptide processing serine protease EPIP precursor by D39V annotation; frameshifted in TIGR4	
Sapep Family Mn^2+^-dependent Dipeptidase/Xaa-His Dipeptidase	SPD_RS10475 SPD_1979 SPV_1979 SP_RS10975 SP_2153	Cleavage of Xaa-His dipeptides (Xaa = a hydrophobic amino acid)	Peptidase M20 family, subfamily M20C; exopeptidase dimerization domain; zinc peptidase domain	
Site-2 Protease Family Protein; Putative Peptidase M50	SPD_RS08385 SPD_1575 SPV_1575 SP_RS08855 SP_1784	Metalloprotease; cleavage of protein transmembrane domains; RIP protease	M50 family of putative membrane-associated zinc metalloproteases; contains a conserved zinc-binding HExxH motif; RIP proteases are involved in a two-step process of cleaving transmembrane proteins	
SprT Family Metalloprotease, putative^	SPD_RS04300 – – SPV_2244 SP_RS04490 SP_0909	Zinc metalloprotease	Contains a conserved zinc-binding HExxH motif found in zinc metalloproteases; locus tag not present in D39 accession number CP000410.2	
Type IV Prepilin Peptidase, putative	SPD_RS08500 SPD_1593 SPV_1593 SP_RS08975 SP_1808	Cleavage of leader peptide from type IV prepilin-like proteins	Gene names and synonyms according to D39V annotation: *cclA, cilC, pilD*	
U32 Family Peptidase	SPD_RS03790 SPD_0704 SPV_0704 SP_RS03920 SP_0801	Unknown	Diverse family with unknown catalytic mechanisms	
U32 Family Peptidase	SPD_RS06710 SPD_1256 SPV_1256 SP_RS07000 SP_1427	Unknown	Diverse family with unknown catalytic mechanisms; noted as incomplete sequence in D39 but not in D39V; incomplete sequence in TIGR4; D39V annotation indicates SPV_1256 to encode a large C1 subunit	
U32 Family Peptidase	SPD_RS06720 SPD_1258 SPV_1258 SP_RS07010 SP_1429	Unknown	Diverse family with unknown catalytic mechanisms; D39V annotation indicates SPV_1258 to encode a large C1 subunit	
YdiL	SPD_RS01935 – - SPV_2157 SP_RS02120 SP_0430	Putative membrane peptidase, hypothetical protein	TIGR4 protein sequence does not indicate homology to any protease or peptidase families; frameshifted in D39	
YhfC Family Intramembrane Metalloprotease	SPD_RS06540 SPD_1223 SPV_1223 SP_RS06820 SP_1391	Metalloprotease	Intramembrane metalloprotease YhfC Family; possess a consensus signature and two additional conserved motifs	
Zinc Protease (Insulinase Family Protein)^	SPD_RS10855 SPD_2052 SPV_2052 SP_RS11360 SP_2225	Metallopeptidase	M16 family peptidase (requires a divalent cation such as zinc)	
ZmpB	SPD_RS03120 SPD_0577 SPV_0577 SP_RS03260 SP_0664	Zinc metalloprotease	Contains a conserved zinc-binding HExxH motif found in zinc metalloproteases	28, 84, 105, 110, 112, 113, 114; 111,
ZmpC	– - – - – - SP_RS00380 SP_0071	Zinc metalloprotease	Not present in D39; Contains a conserved zinc-binding HExxH motif found in zinc metalloproteases	110, 115–117, 119, 120, 118
ZmpD	– - – - – - – - – -	Zinc metalloprotease	Not present in D39 or TIGR4; Contains a conserved zinc-binding HExxH motif found in zinc metalloproteases	27, 110

* National Center for Biotechnology Information (NCBI] gene/nucleotide accession numbers for strains D39 [122] with new locus tags, D39 with old locus tags, D39V [124], TIGR4 [123] with new locus tags, and TIGR4 with old locus tags are NC_008533.2, CP000410.2, CP027540.1, NC_003028.3, and AE005672.3, respectively.

#The D39 and TIGR4 annotations list two genes as *pepF*. The D39V annotation distinguishes these genes as *pepF1* and *pepF2*. Both gene products are approximately 600 amino acids but only share 24% identity in the D39 background.

^These locus tags were identified as encoding proteins with putative protease activity by the deep annotation method employed for D39V [124].

## Aminopeptidase N (PepN)

PepN is a lysyl peptidase that is localized to the bacterial cell wall but does not possess an LPXTG motif [23]. PepN has an inhibitory effect on T cell receptor signaling; however, this inhibition is not due to protease activity by PepN [24]. *S. pneumoniae* deficient in PepN are reduced in nasopharyngeal colonization in mice, reduced in adhesion and invasion of lung A549 cells, cause reduced inflammation in mouse lungs, and cause reduced production of inflammatory cytokines in vivo [25]. It is unclear whether the peptidase function of PepN contributes to the effects. PepN belongs to the peptidase M1-type family. Interestingly, PepN possesses an endoplasmic reticulum aminopeptidase 1-like (ERAP1-like) carboxyl-terminal domain that has been linked to pathways involved not only in metabolism but also in immune functions.

## CAAX Proteases and Bacteriocin-Processing (CPBP) Family Intramembrane Metalloproteases, and Sortases

*S. pneumoniae* possesses numerous putative intramembrane proteases that are related to eukaryotic CAAX enzymes, which are prenyl proteases in the endoplasmic reticulum membrane that cleave CAAX motifs from their respective substrates. For the purpose of brevity, the locus tags for all of these putative proteases are omitted from Table 1. A review of these proteases is described by 26. In short, the CAAX proteases in eukaryotes are involved in the attachment of lipids to proteins, allowing the proteins to be membrane-embedded. In bacteria, the related CPBP proteases are somewhat similar to PrsW (“protease responsible for activating sigma factor W;” Table 1), with general function of regulated intramembrane proteolysis (RIP) as is listed for RseP (“regulator of sigma-E protease;” described in the next section).

Sortases, which act as membrane-localized proteases and transpeptidases, typically recognize the LPXTG motifs on proteins that are to be transported and displayed on the bacterial cell surface. Cleavage usually occurs at the carboxyl-terminus of the target protein, although the LPXTG motif is located on the amino-termini of some proteins such as IgA1 protease, zinc metalloprotease B (ZmpB), ZmpC, and ZmpD [27]. Sortases have been reviewed elsewhere [29].

## Choline-binding Protein D (CbpD) and Regulator of Sigma-E Protease (RseP)

CbpD is a protein that is covalently bound to the phosphorylcholine residues on the cell wall teichoic acids of *S. pneumoniae*. This protein possesses a cysteine, histidine-dependent amidohydrolase/peptidase (CHAP) domain and is responsible in part for peptidoglycan-mediated lysis of subpopulations of pneumococci which results in the release of DNA that can be taken up by neighboring *S. pneumoniae*. Although other pneumococcal proteins are also involved in bacterial cell lysis (LytA for example), the active site cysteine of the CbpD CHAP domain has been determined to be required for lysis, suggesting that the lytic function is dependent on protease/peptidase activity [31]. Lysis of neighboring bacteria, which has been popularly coined microbial fratricide, serves an important function in virulence. Elimination of non-competent bacteria in a population reduces competition for nutrients by the remaining bacteria, improving fitness and survival which are tied to pathogenesis. Moreover, DNA release and subsequent uptake by the remaining pneumococci can introduce new virulence-associated genes to the chromosome. Aside from its role in bacterial lysis, CbpD may possess other virulence functions. *S. pneumoniae* deficient in CbpD are significantly less effective at nasopharyngeal colonization, although the mechanism by which CbpD contributes to colonization is unknown [32].

Related to the ability of CbpD to cause bacterial cell lysis is another pneumococcal protease, the regulated intramembrane proteolysis (RIP) metalloprotease RseP (“regulator of sigma-E,” as named in *Escherichia coli* for its role in activating stress response sigma factors), also known as enhanced expression of pheromone protein (Eep) and other aliases. This intramembrane protease cleaves ComM, a competence protein that is situated in the cell membrane and protects competent *S. pneumoniae* from CbpD-mediated lysis. Unregulated quantities of ComM cause pneumococcal cell shape abnormalities and growth defects; RseP (along with other unidentified factors) controls the quantity of ComM [33].

## Choline-binding Protein G (CbpG)

According to European Molecular Biology Laboratory-European Bioinformatics Institute (EMBL-EBI) InterPro tool analysis of GenBank protein accession number AAF87770.1, CbpG is a trypsin-like serine protease of the peptidase S1, PA clan superfamily of peptidases. CbpG protein sequence indicates that this protein possesses a chymotrypsin-like fold and double beta-barrel structure with a carboxyl-terminal choline-binding domain. An *S. pneumoniae* CbpG-negative mutant exhibited only 25% of wild type ability to colonize the rat nasopharynx, a reduced adherence to nasopharyngeal cells *in vitro*, and a significant reduction in virulence (0% mortality) in infant rat septicemia [32]. The specific mode of action of CbpG in its contribution to adherence and colonization requires that CbpG is in its bacterial surface-bound form via the carboxyl-terminal choline-binding domain [34]. The protease portion of CbpG cleaves casein and fibronectin, and enzyme activity remains intact regardless of the presence or absence of the choline-binding domain [34]. The truncated form of CbpG is released from the bacterial cell and retains protease activity. To date, it is unclear whether the protease activity of the amino-terminal portion plays a role in mediating adherence to host cells, or whether part or all of the amino-terminal portion can provide the adherence link despite the presence of protease. Although the specific mechanism of the proteolytic activity has not been defined at the molecular level in pathogenesis, the observation that the amino-terminal protease-containing domain of CbpG is important for adherence warrants additional investigation. However, a role for this domain in invasive disease is not supported, as truncated CbpG mutants expressing the protease domain but deleted in the choline-binding domain are attenuated [34].

## Caseinolytic Protease P (ClpP)

Clp proteins constitute complexes that use ATP hydrolysis to drive the processing and degradation of misfolded or otherwise defective proteins. Thus, Clp proteins are essential to maintaining homeostasis. Most are part of the heat shock protein 100 (Hsp100) family of stress response proteins, and are also present in plants and animals. Some of these proteins, such as ClpA and ClpX, are the ATP-binding subunits with ATPase activity and recognize protein targets tagged for degradation. ClpA or ClpX (as well as other Clp proteins) can complex with ClpP to form ClpAP or ClpXP protease. The protein constituent of the protease that possesses the enzymatic activity is ClpP, a cytoplasmic serine protease of the S14 peptidase family (MEROPS accession number MER0000474). The active site of ClpP is in the order of serine-histidine-aspartate, a characteristic unique to this family. Individual ClpP proteins assemble to form a homotetradecameric channel through which faulty proteins are transported and cut.

ClpP is required for adherence of *S. pneumoniae* to A549 lung cells in vitro. Pre-incubation of pneumococci with anti-ClpP antibody reduced adherence to A549 cells by over 50% [36]. Whether the reduced adherence is due to blocking a direct interaction of ClpP with the host cell, or causing a ClpP-mediated effect on another unknown pneumococcal protein essential for adherence, is unclear. ClpP is also important for uptake of pneumococci by dendritic cells (DCs) and for pneumococcal survival within activated macrophages [37,38]. Wild type pneumococci induce apoptosis of DCs whereas *clpP* mutant pneumococci are significantly abrogated in induction of apoptosis. Again, whether this virulence characteristic of ClpP is directly owing to the enzymatic activity of ClpP, or is a result of other proteins that were affected by ClpP proteolytic processing, is unknown [37]. Regardless, both active and passive immunization with ClpP is highly protective against invasive *S. pneumoniae* disease [36,39]. The ability to target ClpP as a successful means to protect the host indicates that ClpP is a virulence factor, probably by virtue of its function in maintaining bacterial cell homeostasis through misfolded or defective protein degradation.

The *comCDE* operon encodes genes required for pneumococcal competence, notably the production of the competence-stimulating peptide and recognition of that peptide to induce a signal transduction cascade resulting in the increased ability to take up donor DNA [40]. Genetic disruption of *clpP* causes overexpression of the *comCDE* operon [41,42]. This finding suggests that ClpP is involved in the down-regulation of competence, which was confirmed in transcription repression studies that also demonstrated the necessity for the ClpX ATPase [43]. The direct connection between the proteolytic activity of ClpP and regulation of competence has been demonstrated in *Bacillus subtilis*, in which ClpP degrades the transcription factor ComK [44]. It has also been hypothesized that ClpP is responsible for the degradation of ComX, the sigma factor involved in competence [45]. An interesting similarity between the competence system and the Clp protein complexes is that the *comA* and *comB* gene products form an ATP-binding cassette transporter complex. ComAB recognizes and cleaves the pre-peptide from the competence-stimulating peptide and transports the mature peptide outside the cell [30, 46]. ComA is the peptidase in the complex, cleaving at a glycine doublet via its amino-terminal peptidase domain which belongs to the family of cysteine proteases [47].

ClpP is required for nasopharyngeal colonization, lung infection, and systemic disease in several mouse models of pneumococcal infection using ClpP-negative strains and their parental counterparts. These models included inoculation by intratracheal, intraperitoneal, intravenous, and intranasal routes [38,42,48,49]. Immunization with ClpP increased the survival time of mice infected intraperitoneally with *S. pneumoniae* [49] and provided protection in conjunction with two other pneumococcal proteins in mouse pneumonia and sepsis models [50]. ClpP causes apoptosis of neuroblastoma cells [51]. Interestingly, however, *clpP* has reduced expression in lung and pleural fluid during pneumococcal respiratory infection in mice [52]. Conversely, an increase in expression was quantified for *comB*, one of the two genes encoding the transport apparatus for competence-stimulating peptide ComC. These findings further strengthen the idea that ClpP represses competence [52], although it remains speculative whether a direct cleavage event by ClpP is involved in competence inhibition.

## C3-degrading Protease CppA

Degradation of human complement component C3 by *S. pneumoniae* was demonstrated to be independent of capsule type and likely the result of one or more cell wall-associated proteases produced by the bacterium [53]. Since the discovery of C3 cleavage, at least two pneumococcal proteins have been implicated as having proteolytic activity against this complement constituent. These proteins are CppA and PhtA (PhtA to be discussed later). The direct linkage of C3-degrading activity being attributable specifically to CppA is difficult to find in the standard scientific literature; however, the proteolytic activity has been reported in a patent [54]. Nearly the entire sequence of CppA matches to the glyoxalase/bleomycin resistance protein/dihydroxybiphenyl dioxygenase type superfamily of proteins, which contain beta-alpha-beta(3) motifs characteristic of various types of enzymes with affinities for metal ions [55,61]. The gene encoding CppA was determined to be required for fitness of *S. pneumoniae* in the blood of wild-type mice, but nonessential in sickle cell disease mice [56]. These findings were further confirmed when immunization with CppA protected wild type mice but not sickle cell mice [56]. Additionally, CppA was shown to be required for nasopharyngeal transmission in animal models [57], although it is unknown whether the protease function, or some other attribute, of this protein is involved in transmission.

## Glutamyl Aminopeptidase A (PepA)

PepA is a cytosolic aminopeptidase with preference for glutamate and aspartate residues [58]. Sequence analysis of PepA indicates that it belongs to the M42 family of peptidases, members of which have roles in metabolism and amino acid biosynthesis [61]. Whether PepA possesses a functional role in virulence is not yet known.

## High Temperature Requirement A (HtrA; also DegP)

HtrA is a trypsin-like protease of the peptidase S1C family, protease Do subfamily. Proteases of this family, including HtrA, possess a middle trypsin-like serine protease domain and one or more carboxyl-terminal PSD-95/Dlg/ZO-1 (PDZ) domains that are responsible for substrate protein recognition and/or binding [61]. HtrA is homologous to the Degradation of Periplasmic Proteins (Deg) proteases in *E. coli*, which serve as stress response chaperones and proteases [59]. The presence of a putative amino-terminal signal peptide indicates that HtrA is located on the surface and/or secreted from *S. pneumoniae* [60]. HtrA functions as a molecular chaperone at normal temperatures (28 °C to 37 °C) and as a protease at higher temperatures such as 42 °C [62]. HtrA cleaves non-native derivatives of penicillin-binding protein 2x, a cell wall protein [63], and is important for protection against oxidative stress [64].

The role that HtrA plays in pneumococcal competence is complicated by apparent conflicting results. In one case, *S. pneumoniae* deficient in HtrA were significantly reduced in transformation efficiency, suggesting that HtrA was necessary for competence [64]. In another case, deletion of most of *htrA* or mutation of its putative catalytic residue, had no effect on competence [65]. However, the mutation methods as well as the transformation conditions were different between these two studies. HtrA degrades competence-stimulating peptide [66], although addition of abundant exogenous competence-stimulating peptide does not restore competence [65]. As described above, the competence-stimulating peptide elicits transformation in *S. pneumoniae* as part of a network of competence-related genes [40]. The specificity of HtrA for the competence-stimulating peptide lies at a phenylalanine (nonpolar) residue, and addition of denatured but not intact bovine serum albumin blocks cleavage of the peptide [66]. The complexity surrounding the function of HtrA in competence may be dissected by examination of the temporal nature of competence proteins. *S. pneumoniae* cells in a competent state experience significant decreases in almost all of the competence-related proteins over time, then a stabilization of these proteins [67]. Exceptions to this stabilization are two of the competence protein complexes, ComEA and ComEC, which constitute major membrane components that bind and take up donor DNA. These proteins are nearly depleted by the end of competence [67]. Mutants deficient in HtrA, while significantly reduced in transformation efficiency as was shown previously [64], did not experience ComEA or ComEC degradation, suggesting that HtrA degrades these proteins late in competence [67]. HtrA appears to perform its regulatory function depending upon bacterial culture conditions, as this protease suppresses competence in rich complex medium but not in a chemically-defined medium [68]. Collectively, these studies suggest that HtrA performs the role of a competence regulator at the protein level and that regulation depends in part upon environmental conditions. This idea is supported by the finding that HtrA, specifically its proteolytic active site serine residue, is not able to inhibit competence when coding errors are increased during protein synthesis [69].

In addition to its role in competence, HtrA appears to regulate bacteriocin production. Opaque variants of *S. pneumoniae* produce the bacteriocin, pneumocin MN, whereas transparent variants do not [70]. Opaque variants produce four-fold less message for *htrA*. Deletion of *htrA* in the transparent variants caused an increase in pneumocin MN production. Control of pneumocin MN production by HtrA is accomplished by HtrA limiting the processing and secretion of the bacteriocin peptide pheromone, bacteriocin-like peptide C (BlpC) [71]. Neighboring bacteria sense BlpC, which binds to the histidine kinase BlpH, inducing transcription of the genes in the *blp* locus and resulting in pneumocin MN production; if BlpC export is controlled or limited, then sensing by neighbors and therefore pneumocin MN production is limited [71].

Although HtrA had been well-studied in other species, *S. pneumoniae*-specific HtrA was not documented until less than 20 years ago, when it was identified as having reduced expression in a strain deficient in competence induction and altered cefotaxime susceptibility response regulator/histidine kinase (CiaRH), the two-component signal transduction system which regulates *htrA* [72]. HtrA was further determined to be the CiaRH-controlled protein important for nasopharyngeal colonization [72]. HtrA is important for virulence in mouse pneumonia. Mutants had reduced or nil bacterial loads in lung, blood, spleen, and liver. Likewise, *htrA* mutants caused reduced inflammatory cytokine levels in lung, reduced lung infiltrate, and increased survival [60,64].

## Immunoglobulin A1 (IgA1) Protease

The *S. pneumoniae* IgA1 protease was discovered in 1979 and shown to cleave IgA1 but not IgA2 or other immunoglobulins [19,20]. It was proposed to play a role in mucosal attachment to host cells based on IgA protease activity comparisons between strains isolated from asymptomatic individuals and patients with invasive *S. pneumoniae* infections [73]. IgA1 protease is a cell wall-associated metalloprotease which can be released during late stationary phase of *S. pneumoniae* [74,75]. This protease contains a conserved zinc-binding HExxH motif found in zinc metalloproteases, toward the carboxyl-terminus [75,76]. A distinguishing feature of IgA1 protease that it shares with three other zinc metalloproteases is that its LPXTG motif is present on the amino-terminus instead of the carboxyl-terminus [75,76]. IgA protease appears to be active in multiple forms with masses ranging from approximately 120 to 220 kilodaltons [77]. This protein can split and then recombine to form an active protease with the amino-terminal region binding IgA1 and the carboxyl-terminal region exhibiting protease activity [78].

IgA1 protease cuts the hinge region of the Fc fragment of human IgA1 [79] while leaving the Fab fragments attached to the bacteria [80]. The cleavage site in the hinge region of human IgA1 by streptococcal IgA1 proteases is at a proline-threonine bond [79], although *S. pneumoniae* IgA1 protease (unlike that from other streptococcal species) effectively cleaves at this location when these amino acids are substituted [80,81]. As a result of cleavage, the binding of the Fc portion of IgA1 to *S. pneumoniae* is reduced, and neutrophil phagocytosis of *S. pneumoniae* mediated by capsule-specific IgA1 is inhibited [82]. Moreover, Fab fragment decoration on the bacteria enhances their attachment to host epithelial cells via the receptor for platelet-activating factor (rPAF), promoting pathogenesis [83].

IgA1 protease is required for virulence in a mouse model of intranasal infection. Intranasal infection with an IgA1 protease-negative *S. pneumoniae* strain resulted in significantly increased mouse survival compared to infection with the parent strain [84]. As part of a protein vaccine, IgA1 protease provided protection against pneumococcal pneumonia in mice with decreased bacterial loads recovered from blood, lungs, and nares [85].

## Murein (Peptidoglycan) Peptidases DacA and DacB

Maintenance of bacterial cell shape and peptidoglycan turnover during division are important to the viability and fitness of *S. pneumoniae*, which in turn renders these activities important for virulence potential. Moreover, peptidoglycan is recognized by host receptors such as Toll-like receptors (TLRs) and NOD-like receptors (NLRs) which mediate immune responses to pathogens. Bacterial carboxypeptidases function to cleave amino acids from the ends of the peptidoglycan stem pentapeptides, and endopeptidases cleave within peptides of the stems or the cross-bridges. D-alanyl-D-alanine carboxypeptidase (DacA; also known as penicillin-binding protein 3) is a bacterial cell wall-attached carboxypeptidase that has been extensively characterized [86–88]. DacA cleaves the bond between the two D-alanine residues of the stem peptide, then a second carboxypeptidase, DacB (also known as LdcB for “L,D-carboxypeptidase B”), cleaves the next bond remaining (between L-lysine and D-alanine) [89]. Although protein sequence analysis indicates it to be a putative D,D-carboxypeptidase, DacB has instead been verified to be an L,D-carboxypeptidase [86,89–91].

DacA and DacB were investigated for their possible roles in virulence in a mouse model of intranasal inoculation leading to pneumonia and sepsis [86]. Pneumococcal mutants with *dacA* deletion produced lung and blood infection similar to the parent strain, although onset of sepsis was slightly delayed. In contrast, mutants with *dacB* or *dacA/dacB* deletions were significantly attenuated in virulence, with marked delays in spread to lungs and subsequently the bloodstream [86].

## Neutral Endopeptidase O (PepO)

Neutral endopeptidase O (PepO) belongs to the M13 family of peptidases, a wide group of metallopeptidases in eukaryotes and bacteria, which possess cytoplasmic, transmembrane, and extracellular domains with the carboxyl-terminal extracellular domain containing the catalytic site [61]. In *S. pneumoniae*, PepO has been found to be released into the extracellular medium beginning in early logarithmic phase and its secretion increases as bacterial growth progresses [92]. PepO binds to and cleaves plasminogen to generate plasmin when in the presence of urokinase-type plasminogen activator (uPA), resulting in cleavage of complement component C3b [92]. Moreover, PepO reduces C3b deposition on *S. pneumoniae* [92,93]. PepO also binds to fibronectin, but the degree of binding appears to be strain-specific as one strain of *S. pneumoniae* deficient in PepO experienced a ~ 75% loss of binding whereas another strain did not have significant loss of binding to fibronectin [92]. The ability of PepO to activate plasminogen in the presence of uPA resembles the binding of other *S. pneumoniae* proteins, most notably enolase, but also CbpA/PspC/SpsA and translation elongation factor Tu (Tuf), to plasminogen [35, 92, 94 .

Exogenously added PepO reduces adhesion and invasion of lung cells by *S. pneumoniae*, suggesting that PepO is involved in attachment to host cells [92]. Complement component C1q enhances pneumococcal attachment to host cells, and PepO also binds to C1q; this binding can occur with secreted or cell surface-bound PepO [93]. Pre-treatment of serum with PepO inhibits the ability of aggregated immunoglobulin G (IgG) to activate the classical complement cascade at the C1q step, and PepO can activate complement to similar levels as those of IgG as determined by C1q and C3 deposition [93]. PepO also binds to the complement inhibitor C4b-binding protein (C4BP) and this interaction is noncompetitive with PepO binding to C1q. It has been proposed that secreted PepO, bound or not bound by C1q, binds and inactivates C4BP, leading to complement consumption as a result of unregulated activation. At the same time, surface-exposed (non-secreted) PepO bound by C1q promotes *S. pneumoniae* attachment to host epithelial cells. In this model, *S. pneumoniae* would evade complement deposition while exploiting complement components to colonize and invade host cells [93]. On the other hand, it has also been shown that PepO enhances phagocytosis by macrophages, indicating that PepO aids the host defense against *S. pneumoniae* [95,96].

PepO stimulates Toll-like receptors 2 and 4 (TLR2 and TLR4), which recognize bacterial patterns and trigger intracellular signaling cascades resulting in the production of inflammatory cytokines [95,97]. PepO increases the production of cytokines in mouse lungs, and this increase is abrogated in TLR4-negative mice with a lesser effect observed in TLR2-negative mice [97]. It has been proposed that exogenous PepO could be used as an immunotherapeutic to treat *S. pneumoniae* infections [95]. Whether PepO is a bona fide virulence factor [92,93] or not [95–97] is not fully resolved.

## Pneumococcal Histidine Triad Protein A (PhtA; also PhpA)

Following the finding that *S. pneumoniae* produced C3-degrading activity [53], Hostetter identified two proteases that potentially comprise such activity [98]. One of these proteins was CppA (described above) and the other was a 20 kDa fragment of a larger 79 kDa protein. This protein was named pneumococcal histidine protein A, or PhpA [99], but is more commonly known as PhtA. PhtA is one of at least four known histidine triad proteins in *S. pneumoniae*, and these proteins have been shown to have high affinity for zinc. PhtA possesses a putative signal sequence, suggesting its localization to the cell surface [100].Deletion of PhtA and the other three Pht proteins results in increased C3 binding to *S. pneumoniae*; however, single deletion of PhtA has no effect on C3 deposition, suggesting that these proteins have interdependent roles regarding effect on complement [101,102]. Immunization of mice with PhtA significantly improved survival following intranasal infection [99], and intraperitoneal infection with serotypes 6A and 6B [100], of *S. pneumoniae*.

## Pneumococcal Protease A (PrtA)

PrtA is a cell wall-associated serine protease of the S8 family of peptidases, which cleave amino-terminal leader sequences from lantibiotics [103,61]. Lantibiotics are bacteriocin peptides that are cidal to competing bacteria. Systemic infection of mice with a *prtA* mutant strain of *S. pneumoniae* resulted in significantly longer survival times compared to infection with the parent strain [103]. The gene encoding PrtA is upregulated in blood following systemic infection in mice and a PrtA-deficient strain is significantly attenuated in an intranasal infection model. PrtA appeared to be required for movement to the mouse lung following intranasal infection, as the *prtA* mutant colonized the nasopharynx similar to its parent strain but had reduced colony-forming units in the lung [104]. This effect was found to be strain-specific as PrtA was not required for full virulence in all strain backgrounds tested [60,104]. Nonetheless, PrtA interacts with collagen type IV and plasminogen, suggesting that its possible cleavage of these substrates could contribute to tissue invasion and breach into the bloodstream [105]. In contrast, PrtA cleaves human apolactoferrin to generate lactoferricin-like peptide, which acts as a cationic antimicrobial peptide and kills *S. pneumoniae* [106]. The cleavage of apolactoferrin to promote killing of *S. pneumoniae* does not support the notion that PrtA is a virulence factor, although there may be another unidentified mechanism to promote virulence that would require this cleavage.

## Signal Peptidases

Signal peptidases are important for the processing and display, secretion, and/or function of proteins produced by bacteria. Type I signal peptidases are serine peptidases with catalytic dyads comprised of serine and lysine, that cleave amino-terminal leader peptides from many proteins and have been well-described for the canonical Sec machinery in bacterial secretion systems. Type II signal peptidases are aspartate peptidases that recognize a lipobox sequence of four amino acids in pro-lipoproteins, cleaving the lipoproteins to their mature forms.

Signal peptidase I (leader peptidase LepB) in *S. pneumoniae*, a single-pass transmembrane protein with an extracellularly exposed carboxyl-terminus [61], is required for bacterial viability [107]. Lipoprotein signal peptidase (LspA) is a transmembrane type II signal peptidase which cleaves amino-terminal signal peptides containing lipobox sequences with a conserved cysteine from pro-lipoproteins [108]. Lipoproteins are anchored to the exterior of the pneumococcal cell membrane following modification by a lipoprotein diacylglyceryl transferase, Lgt, then they are cleaved by LspA. Proteomic analysis of the *S. pneumoniae* surface and extracellular contents showed that Lsp is necessary for the maintenance of cell surface localization for some proteins and for overall protein integrity for others [109]. Interestingly, in the absence of Lgt, LspA was shown to process specific proteins despite the lack of post-translational modification by Lgt. Furthermore, the lipoprotein SlrA was cleaved of its signal peptide in the absence of both Lgt and LspA, indicating that *S. pneumoniae* may possess an alternate lipopeptidase [109].

Lipoproteins comprise subunits of ABC transporters in *S. pneumoniae* which serve a variety of functions. For example, a manganese uptake ABC transporter lipoprotein helps protect *S. pneumoniae* from oxidative stress. Deletion of the gene encoding LspA causes a failure in cleavage of the pro-lipoprotein of this ABC transporter and impaired protection from oxidative stress. Loss of LspA results in reduced virulence in mouse pneumonia and septicemia models of infection [108].

## Zinc Metalloprotease B (ZmpB)

The gene encoding ZmpB is present in all strains of *S. pneumoniae* tested [110]. ZmpB contains an amino-terminal LPXTG motif and is tethered to the cell surface with the carboxyl end exposed. Two studies of ZmpB function related to bacterial physiology yielded conflicting data. One study reported that *zmpB* mutants of an encapsulated type 4 strain were unable to autolyze, formed long chains, lacked surface expression of specific choline-binding proteins comcomitant with intracellular entrapment of autolysin (LytA) and CbpA/PspC/SpsA, and were reduced in transformation efficiency [111]. In contrast, another group reported that none of these effects occurred in their *zmpB* mutants in a non-encapsulated strain, which was then tested and confirmed in a type 4 strain [28]. The latter group ascertained that the *zmpB* mutant strain used in the first study was actually a Viridans group streptococcus and was not derived from the parent *S. pneumoniae* strain [28].

ZmpB is required for virulence in mouse models of pneumococcal pneumonia and septicemia. Mucosal immunization with ZmpB was protective against pneumonia and septicemia potentially via T helper 1 (Th1) and Th17 responses [112]. Additionally, mice infected with *zmpB* knockout strains had significantly increased survival compared to those infected with the parent strains [84,113]. Bacterial quantities in the blood were significantly reduced following infection with a *zmpB* mutant, and the lack of an effect in complement C3-negative mice indicated that ZmpB does not affect complement-mediated opsonization. Reduced levels of tumor necrosis factor-alpha (TNF-alpha) in the lungs suggested that ZmpB could be required for inflammatory events leading to *S. pneumoniae* translocation from the lungs to the blood [113]. Since ZmpB interacts with collagen type IV, likely through proteolytic cleavage, perhaps ZmpB compromises the integrity of lung cell collagen type IV to invade the blood [105].

Despite its interaction with collagen type IV, ZmpB was not required for intranasal colonization or bacterial survival in lungs, suggesting that this protease is not required for bacterial adhesion to host cells [113]. However, competitive inhibition by recombinant ZmpB or ZmpB antibodies reduces bacterial binding to lung cells *in vitro* [112]. The strain background and presence or absence of other zinc metalloproteases with possible functional redundancy could be contributing factors, as well as the sequence variations between *zmpB* genes among different strains of *S. pneumoniae* [114].

## Zinc Metalloprotease C (ZmpC)

The gene encoding ZmpC is present in only about 18–24% of *S. pneumoniae* clinical strains [110,115,116]. As for ZmpB, ZmpC is sorted to the cell surface via an amino-terminal LPXTG. Strains that harbor *zmpC* are associated with more severe clinical disease [115], and ZmpC has been shown to be required for virulence in a mouse model of pneumonia [117]. In contrast, ZmpC-negative *S. pneumoniae* cause increased mortality and increased bacterial loads in the blood and brains of mice infected via the intravenous route, which was suggested to be a strategy by ZmpC-positive *S. pneumoniae* to extend the life of the host to benefit bacterial survival [118]. ZmpC causes ectodomain shedding of syndecan-1, a heparin sulfate proteoglycan present on epithelial cells, from mouse mammary gland epithelial cells [119]. This shedding event is significantly reduced by inhibitors of metalloproteinase activity, Ilomastat and TNF-alpha protease inhibitor 1 (TAPI-1), indicating that the protease activity of ZmpC is responsible for ectodomain shedding [119]. Direct cleavage of syndecan-1 ectodomains by semi-purified native ZmpC was also demonstrated, confirming the protease activity [119]. Furthermore, ZmpC causes ectodomain shedding of membrane-associated mucin MUC16 from human tracheobronchial, corneal, and conjunctival cells [120]. ZmpC also cleaves and activates human matrix metalloproteinase 9 (MMP-9) [117]. Collectively these results show that ZmpC is a virulence factor with several host substrate targets that are important for structural integrity, mucosal protection, and tissue remodeling.

Another host target of ZmpC is the neutrophil. ZmpC cuts P-Selectin Glycoprotein-1 (PSGL-1) present on human neutrophils, inhibiting its ability to bind to P-Selectin on endothelial cells [116]. By reducing the neutrophil’s ability to bind to endothelial cells, initial neutrophil rolling movement along the blood vessel lumen is interrupted and thus extravasation is inhibited. Subverting neutrophil extravasation, therefore, would also cause a decrease in phagocytic clearance of *S. pneumoniae*. Indeed, neutrophil rolling was inhibited by ZmpC *in vitro*, and reduced neutrophils were recovered from mouse lung lumen while neutrophil numbers were unaffected in the lung interstitial tissue *in vivo* during the early stage of infection by a *zmpC* mutant of *S. pneumoniae* [116].

## Zinc Metalloprotease D (ZmpD)

The presence of ZmpD in *S. pneumoniae* strains is variable, with about half of strains possessing *zmpD* in the chromosome [110]. The gene encoding ZmpD appears to be acquired from genetic recombination events between members of the Mitis group of streptococci [27]. When present, *zmpD* is located adjacent to the gene which encodes IgA1 protease (*zmpA*, or *iga*) and its presence is hypothesized to be a result of a duplication event [27]. The *zmpA/zmpD, zmpB*, and *zmpC* loci are distinct from each other and exhibit high sequence variability in *S. pneumoniae* [27]. ZmpD shares a similar cellular locale as the other zinc metalloproteases in that this enzyme has an amino-terminal LPXTG motif and is attached to the surface of *S. pneumoniae*. The specific functional role of ZmpD in pathogenesis is unknown.

## Figures and Tables

**Figure 1.**
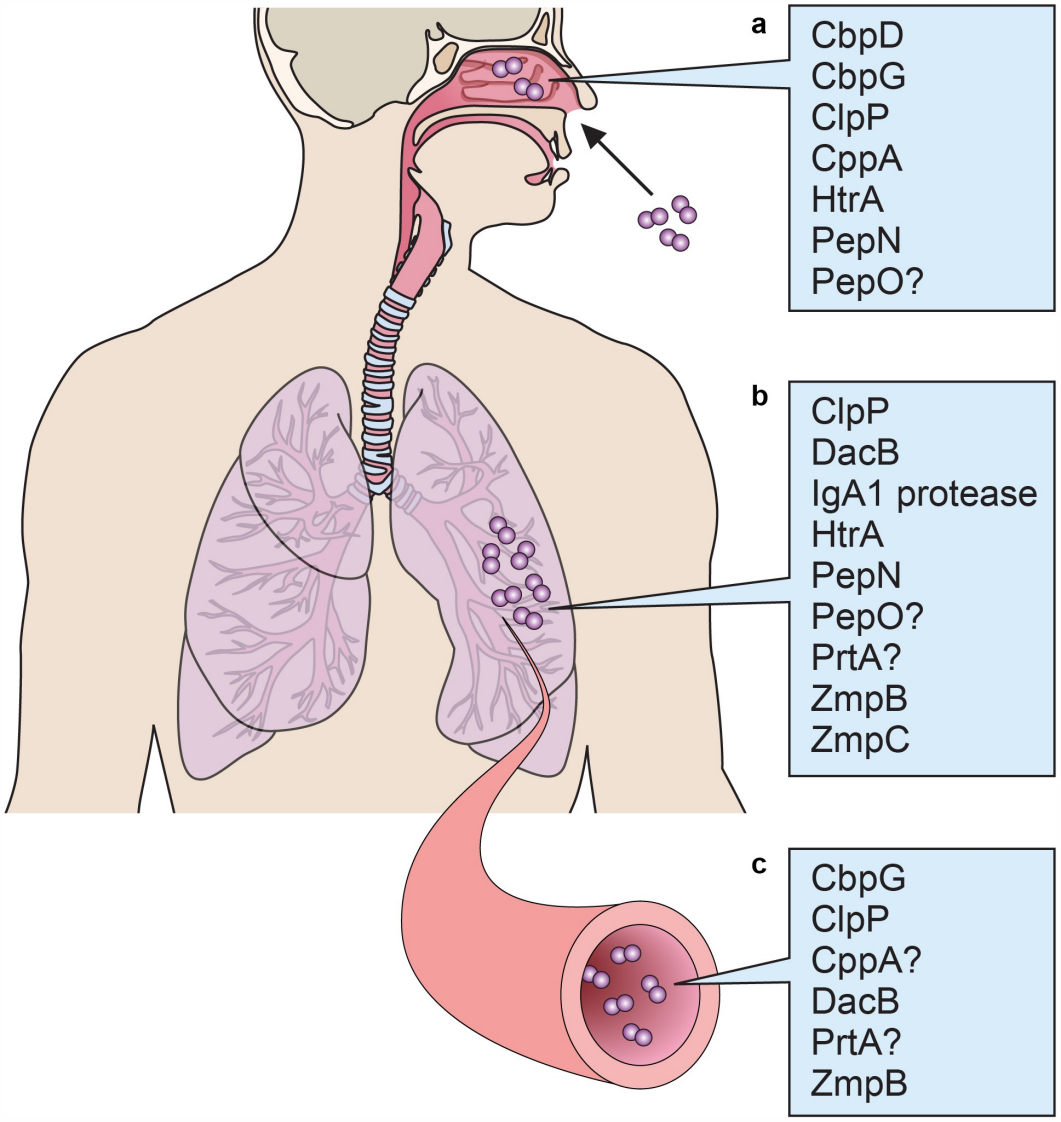
Pneumococcal proteases implicated in A) colonization, B) pneumonia, and C) septicemia

**Figure 2.**
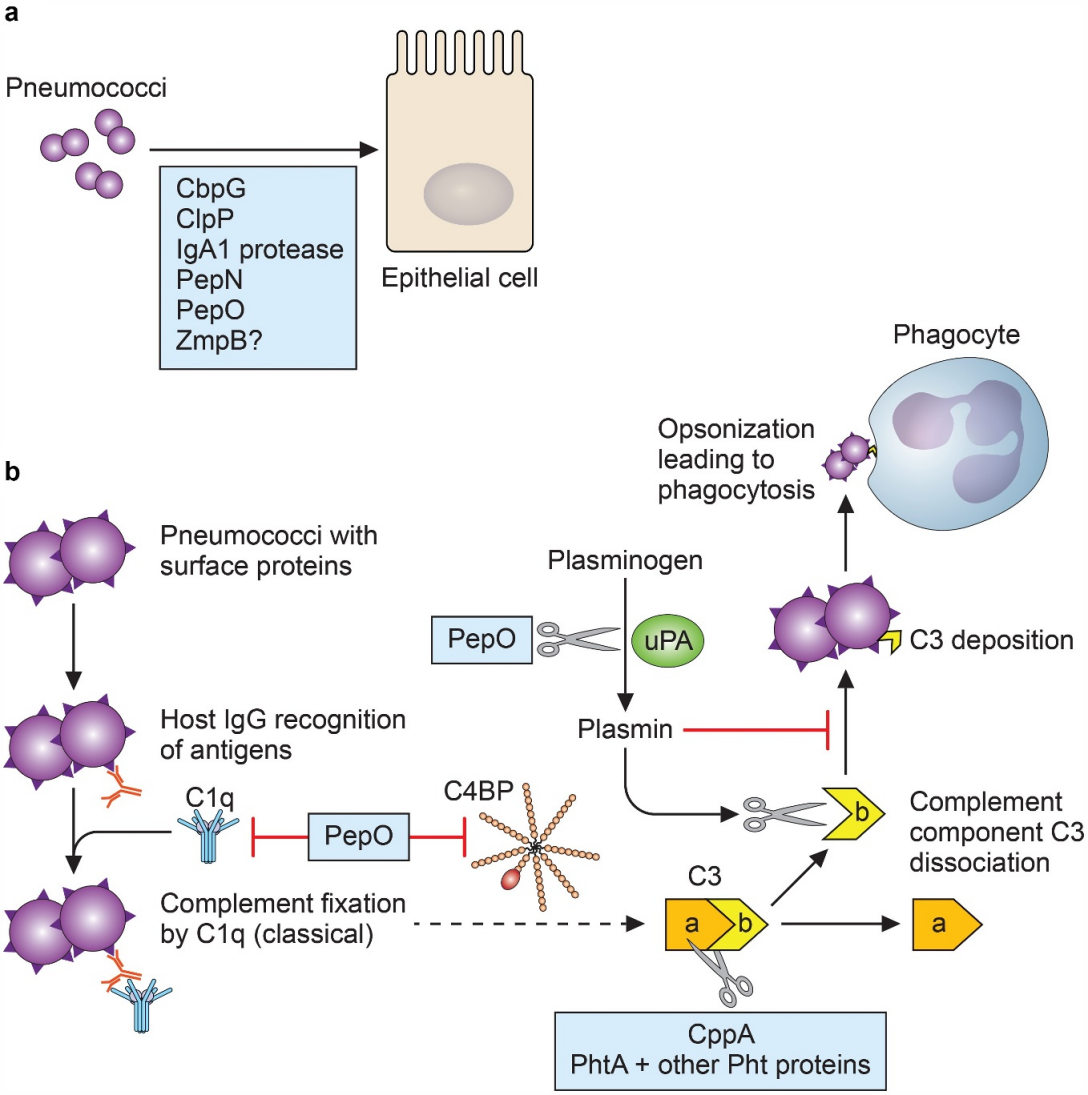
Roles of pneumococcal proteases at the host cellular level. (a) At least 5 proteases are involved in attachment to nasopharyngeal or lung epithelial cells. (b) Pneumococcal proteases can inhibit complement deposition, as depicted by the abridged classical complement pathway. CppA and histidine triad proteins degrade complement component C3, which is important for pathogen recognition by phagocytes. PepO can bind to C1q, but can also act as a complement activator and promote premature consumption by inhibiting the regulatory inhibitor C4BP. In the presence of uPA, PepO cleaves plasminogen to the proteolytically active plasmin, and plasmin prematurely degrades C3b and inhibits its deposition on *S. pneumoniae*. Since C3b is necessary for binding to complement receptor on phagocytes such as neutrophils (opsonization), inhibition of C3b deposition contributes to immune evasion

## Summary: Putting the Pieces Together

In terms of pathogenesis, *S. pneumoniae* colonizes the nasopharynx and then spreads to other sites depending on a variety of bacterial and host factors. Pneumococcal proteases with confirmed or tentative functions in colonization (including transmission) are shown in Figure 1. It is not surprising to find that surface choline-binding proteins would be involved in colonization, as these types of proteins have been shown to be important for adherence to host cells. ClpP and HtrA, however, are proteases with classical protein processing or chaperone functions for other pneumococcal proteins. Do these proteases directly cleave host proteins, or do they possess more complex post-translational regulatory functions involving other pneumococcal factors?

Following colonization, the two pneumococcal diseases that are best represented in protease function studies to date are pneumonia and septicemia (Figure 1). ClpP is implicated for both of these diseases, and HtrA is implicated in pneumonia; the question of direct impact on host proteins or indirect effects of these proteases remains. IgA1 protease and ZmpB, however, have less nebulous roles. IgA1 protease directly cleaves human IgA1, which is present at mucosal sites such as lung epithelium, leaving the Fc portions of IgA1 molecules cut and removed from the Fab fragments that bind the pneumococci. ZmpB cleaves collagen, an important lung tissue component, and this function has been suggested to contribute to its role in blood infection by the breach of barriers resulting from collagen cleavage. An interesting item of note is that CbpG has thus far been determined to be necessary for colonization as well as septicemia, perhaps due in part to its fibronectin-cleaving ability. CbpG, unlike ClpP which has also been reported to play roles in both colonization and invasive disease, is not ascribed the function of a processor of bacterial proteins. A protease such as ClpP might be expected to be involved at all stages of colonization and infection by nature of its regulatory function for the bacteria; however, a protease such as CbpG might be a different type of moonlighting protein with multiple functions, or alternately expressed depending on niche conditions. Indeed, at the cellular level, the choline-binding domain of CbpG contributes to pneumococcal attachment to epithelial cells (Figure 2). IgA1 protease cleavage of IgA1 also contributes to host cell binding, as pneumococci with bound Fab fragments attach at epithelial rPAF.

Aside from interactions at the host cell membrane, other molecular events that impact pneumococcal pathogenesis are the complement pathways [121]. One of the main outcomes of classical complement activation is the opsonization and phagocytosis of bacteria by host immune cells, and this outcome depends on the successful deposition of complement component C3b on the bacteria. Proteases that thwart this deposition are CppA, PepO, and PhtA in combination with the other Pht proteins (Figure 2).

Pneumococcal proteases are prime examples of the interconnectivity of bacterial biochemistry and pathogenesis. Most of the proteases and peptidases discussed herein serve essential functions in the maintenance of cellular functions through the cleavage of proteins as normal cellular processes, in response to environmental stressors such as a specific host niche, or as intra- and inter-species competition. The known and potential detrimental effects of these proteases on the host are increasingly becoming elucidated, in great part attributable to data mining of sequence information and proteomics tools.

## Data sharing

Data sharing is not applicable to this article as no new data were created or analyzed in this study.
